# Should we hail the Red King? Evolutionary consequences of a mutualistic lifestyle in genomes of lichenized ascomycetes

**DOI:** 10.1002/ece3.8471

**Published:** 2022-01-11

**Authors:** Claudio G. Ametrano, H. Thorsten Lumbsch, Isabel Di Stefano, Ek Sangvichien, Lucia Muggia, Felix Grewe

**Affiliations:** ^1^ Grainger Bioinformatics Center and Negaunee Integrative Research Center, Science and Education Field Museum of Natural History Chicago Illinois USA; ^2^ Department of Biology Faculty of Science Ramkhamhaeng University Bangkok Thailand; ^3^ University of Trieste Trieste Italy

**Keywords:** coevolution, comparative genomics, evolutionary rate, lichenized fungi, Pyrenulales, Trypetheliales

## Abstract

The Red Queen dynamic is often brought into play for antagonistic relationships. However, the coevolutionary effects of mutualistic interactions, which predict slower evolution for interacting organisms (Red King), have been investigated to a lesser extent. Lichens are a stable, mutualistic relationship of fungi and cyanobacteria and/or algae, which originated several times independently during the evolution of fungi. Therefore, they represent a suitable system to investigate the coevolutionary effect of mutualism on the fungal genome. We measured substitution rates and selective pressure of about 2000 protein‐coding genes (plus the rDNA region) in two different classes of Ascomycota, each consisting of closely related lineages of lichenized and non‐lichenized fungi. Our results show that independent lichenized clades are characterized by significantly slower rates for both synonymous and non‐synonymous substitutions. We hypothesize that this evolutionary pattern is connected to the lichen life cycle (longer generation time of lichenized fungi) rather than a result of different selection strengths, which is described as the main driver for the Red Kind dynamic. This first empirical evidence of slower evolution in lichens provides an important insight on how biotic cooperative interactions are able to shape the evolution of symbiotic organisms.

## Graphical abstract

Lichens are a stable, mutualistic relationship of fungi and cyanobacteria and/or algae, which originated several times independently during the evolution of fungi. Our results show that independent lichenized clades are characterized by significantly slower rates for both synonymous and non‐synonymous substitutions, as predicted by the Red King dynamic for mutualistic relationships. We hypothesize that this evolutionary pattern is connected to the lichen life cycle (longer generation time of lichenized fungi) rather than a result of different selection strengths on specific genes in lichenized fungal taxa.

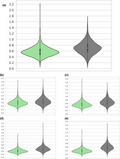

## 1 INTRODUCTION

Coevolution can substantially shape the evolution of organisms involved in intimate ecological connections that range from antagonistic to mutualistic relationships. In its essence, coevolution is a reciprocal evolutionary change induced by interacting species (Thompson, 2014). Possibly, every biotic interaction within the food web involves a certain degree of interdependence resulting in coevolutionary patterns, as any change in a species will influence one or more connected species. If the relationship is tight enough, and the reciprocally induced evolutionary changes last long enough, coevolutionary effects can become apparent. In this context, Van Valen's (1973, 1977) Red Queen dynamic described how biotic interactions can influence evolution. This hypothesis was initially formulated to explain extinction patterns recurring in fossil records of major taxa, but was later extended by the author emphasizing the importance of competitive biotic interactions in a macro‐evolutionary framework. In this hypothesis, coevolution was described as an evolutionary action–reaction cycle, which is characterized by the fluctuations of the relative fitness of two antagonist species. This cycle leads to an arms race regulated by natural selection that eventually accelerates evolutionary rates. Many authors further revised the Red Queen dynamic theory (Brockhurst et al., 2014; Strotz et al., 2018) broadening its original meaning (Morran et al., 2011), confirming (Kerfoot & Weider, 2004), or challenging it (Gokhale et al., 2013; Wei & Kennett, 1983); some of these studies used model simulations (Dercole et al., 2010; Rabajante et al., 2015), or experimental systems (Decaestecker et al., 2007; Paterson et al., 2010), at different organizational (e.g., community, population), temporal, and taxonomic scales (Finnegan et al., 2008; Liow et al., 2011).

The incredibly diversified literature inspired by Van Valen's original hypothesis resulted in a wide concept of the Red Queen dynamic that will be used in this study: coevolution as a driving force that can accelerate evolution (Delaye et al., 2018; Pal et al., 2007; Paterson et al., 2010) and/or modify the selective pressure acting on the coevolving species and their genes (Ejsmond & Radwan, 2015). Though abiotic interactions play a prevalent role as a selective constraint at the largest time and spatial scales (Benton, 2009, 2010; Venditti et al., 2010), biotic interactions can also have a relevant role in stable environments, for long‐lasting, specific associations, such as symbioses. Evidence of biotic relationships as an important long‐term selective force was found in host–parasite interactions, such as a New Zealand snail and its trematode parasites (Dybdahl & Lively, 1998), a plant–fungus association (Thrall et al., 2012), and a bacteria–ant association (Degnan et al., 2004, 2005).

In contrast to the accelerated evolution in host–parasite interactions due to the Red Queen dynamic, the so‐called Red King dynamic (Bergstrom & Lachmann, 2003) hypothesizes slower evolutionary rate as beneficial for mutualistic interactions in relevant classes of mutualistic interactions (Veller et al., 2017). Although empirical evidence for the Red King dynamic is still lacking, theoretical studies modeled Red Queen/King dynamics, evaluating parameters such as mutation rate, population size, selection strength, and generation time to understand what conditions can favor a slower evolving symbiont in mutualistic symbioses (Damore & Gore, 2011; Gao et al., 2015; Gokhale & Traulsen, 2012; Veller et al., 2017).

Molecular evolutionary rate measurements (e.g., nucleotide substitution rates) have been extensively used to test relevant evolutionary hypotheses involving lifestyles (Bromham et al., 2013), to compare large taxonomic groups (Buschiazzo et al., 2012; De la Torre et al., 2017; Wang et al., 2010), or to identify conditions likely responsible for rate shifts (Lanfear et al., 2013). Mutualistic symbioses have been investigated to test evolutionary hypotheses using substitution rates (Arab et al., 2020; Rubin & Moreau, 2016), but attention has been rarely focused on the lichen symbiosis (Lumbsch et al., 2008; Lutzoni & Pagel, 1997; Zoller & Lutzoni, 2003). These lichen studies used multi‐gene datasets, but no study so far addressed differences in substitution rates in lichens using genome‐scale data.

The lichen symbiosis is a stable, successful mutualistic association between at least one fungus (the mycobiont) and one or several photosynthetic partners (green algae and/or cyanobacteria: the photobionts). However, the definition of the lichen symbiosis ranged from a controlled parasitism (Ahmadjian, 1993) to mutualism, and it is still subjected to relevant extensions and revisions (Hawksworth & Grube, 2020). These symbioses developed multiple times independently along the evolutionary history of fungi (Schoch et al., 2009); moreover, the mycobiont is—with rare exceptions—an obligate symbiont, whereas the photobiont is usually not entirely dependent on the mycobiont for survival (Nash, 2008; Wedin et al., 2004). For these reasons, lichenized fungi are a suitable system to explore possible genomic consequences of a mutualistic lifestyle.

We are here using genome‐scale data to test three specific hypotheses: (i) The evolutionary rate of lichen mycobionts differs from the rates of non‐lichenized fungi; (ii) this change in evolutionary rates is due to a different selective pressure acting on mycobionts in comparison with non‐lichenized fungi; and (iii) specific genes are under positive selection in a scenario of general slower or faster evolution.

## 2 MATERIALS AND METHODS

### 2.1 Taxon sampling

**FIGURE 1:**
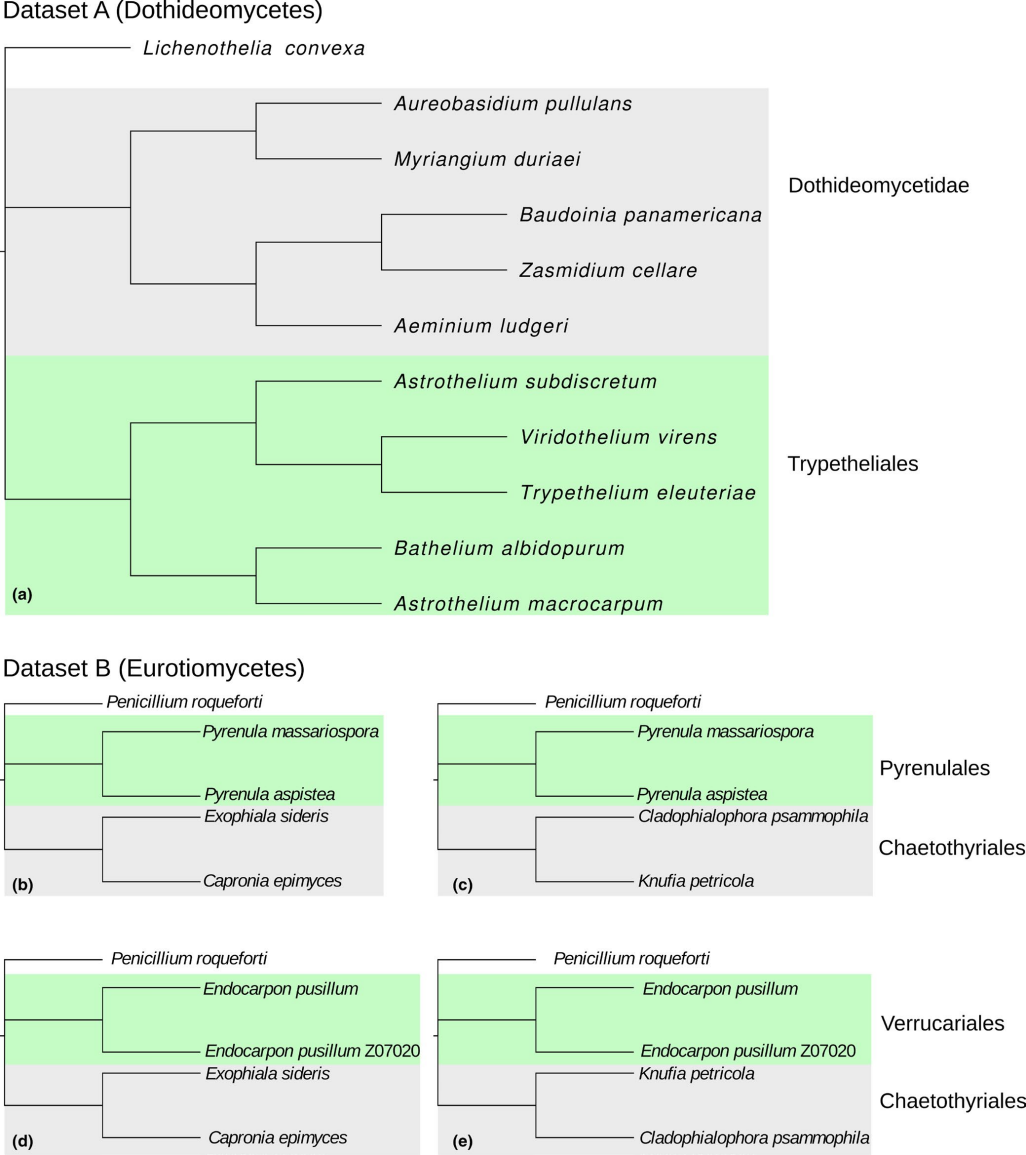
Constrained topologies used to run the PAML rate analyses. (a) Dataset A (Dothideomycetes); (b–e) the four topologies used to compare the two lichenized clades to the non‐lichenized clade in dataset B (Eurotiomycetes). Lichen clade in green, non‐lichenized in gray

A total of eight lichen‐forming fungal species and 11 non‐lichenized fungal species were included in this study. Two datasets, corresponding to two independent lichenization events, which occurred in two Ascomycota classes, were prepared. In dataset A (Dothideomycetes), four lichenized species belonging to Trypetheliales (*Astrothelium macrocarpum*, *A*. *subdiscretum*, *Bathelium albidoporum*, and *Trypethelium eluteriae*) were sequenced in this study from mycobiont cultures; *Viridothelium virens* was added from the NCBI Assembly Database (https://www.ncbi.nlm.nih.gov/assembly). The genome comparisons were performed using an equal number of assemblies from the subclass Dothideomycetidae, which is the most closely related clade to Trypetheliales with genomic resources publicly available. From this clade, *Aeminium ludgeri*, *Aureobasidium pullulans*, *Baudoinia panamericana*, *Myriangium duriaei*, and *Zasmidium cellare* assemblies were retrieved from the NCBI Assembly Database. *Lichenothelia convexa* (Ametrano et al., 2019) was used as outgroup. In dataset B (Eurotiomycetes), two lichenized species belonging to the order Pyrenulales (*Pyrenula aspistea*, *P*. *massariospora*) were sequenced in this study from mycobiont cultures and compared with *Exophiala sideris* and *Capronia epimyces*, and with *Knufia petricola* and *Cladophialophora psammophila*, publicly available on NCBI. The same two couples of non‐lichenized fungi from Chaetothyriales were also compared with two samples of the lichenized species belonging to Verrucariales *Endocarpon pusillum*, retrieved from the NCBI Assembly Database. *Penicillium roqueforti* was used as outgroup. Accession numbers, taxonomic information, and references are listed in Table 1. Outgroup samples were used to clarify the phylogenetic relationship of samples, rerooting the inferred trees (Figure S1), and in the polytomy necessary to identify the constrained trees used for rate analyses as unrooted (Figure 1). In both datasets, an equal number of samples in lichenized and non‐lichenized comparisons were used, in order to avoid a possible node‐density bias (Hugall & Lee, 2007; Venditti et al., 2006).

**TABLE 1:** Genome assemblies with taxonomy and references

Accession number	Sample Name	Class	Order	Reference
GCA_021030915.1	*Astrothelium macrocarpum*	Dothideomycetes	Trypetheliales	This study
GCA_021030935.1	*Astrothelium subdiscretum UBN165*	Dothideomycetes	Trypetheliales	This study
GCA_021031095.1	*Bathelium albidoporum*	Dothideomycetes	Trypetheliales	This study
GCA_021030925.1	*Trypethelium eluteriae*	Dothideomycetes	Trypetheliales	This study
GCA_010094025.1	*Viridothelium virens*	Dothideomycetes	Trypetheliales	Haridas et al. (2020)
GCA_000338955.1	*Baudoinia panamericana* UAMH 10762	Dothideomycetes	Capnodiales	Ohm et al. (2012)
GCA_000721785.1	*Aureobasidium pullulans* EXF‐150	Dothideomycetes	Dothideales	Gostinčar et al. (2014)
GCA_004216415.1	*Aeminium ludgeri*	Dothideomycetes	Capnodiales	Trovão et al. (2019)
GCA_010093895.1	*Myriangium duriaei* CBS 260.36	Dothideomycetes	Myriangiales	Haridas et al. (2020)
GCA_010093935.1	*Zasmidium cellare* ATCC 36951	Dothideomycetes	Capnodiales	Haridas et al. (2020)
GCA_021030975.1	*Lichenothelia convexa* L1844	Dothideomycetes	Lichenotheliales	Ametrano et al. (2019)
GCA_021030945.1	*Pyrenula aspistea*	Eurotiomycetes	Pyrenulales	This study
GCA_021030905.1	*Pyrenula massariospora*	Eurotiomycetes	Pyrenulales	This study
GCA_000464535.1	*Endocarpon pusillum* Z07020	Eurotiomycetes	Verrucariales	Wang et al. (2014)
GCA_000611755.1	*Endocarpon pusillum*	Eurotiomycetes	Verrucariales	Park et al. (2014)
GCA_000585535.1	*Cladophialophora psammophila* CBS 110553	Eurotiomycetes	Chaetothyriales	Teixeira et al. (2017)
GCA_000585565.1	*Capronia epimyces* CBS 606.96	Eurotiomycetes	Chaetothyriales	Teixeira et al. (2017)
GCA_000835395.1	*Exophiala sideris*	Eurotiomycetes	Chaetothyriales	Teixeira et al. (2017)
GCA_002319055.1	*Knufia petricola*	Eurotiomycetes	Chaetothyriales	Teixeira et al. (2017)
GCA_001599855.1	*Penicillium roqueforti*	Eurotiomycetes	Eurotiales	An et al. (2009)

Note

Assemblies produced for this study are in bold. Dothideomycetes: dataset A. Eurotiomycetes: dataset B.

### 2.2 Fungal cultures, DNA extraction, and sequencing

Fungal strains were isolated at Ramkhamhaeng University by Ek Sangvichien. Strains were subcultured on malt–yeast extract until the mycelia grew to a sufficient biomass for DNA extraction.

DNA of all cultures was isolated using the ZR Fungal/Bacterial DNA MiniPrep Kit (Zymo Research), converted into libraries with the KAPA HyperPrep Kit (KAPA Biosciences), and sequenced at the University of Illinois at Chicago Research Resource Center on Illumina's NextSeq Platform. High‐molecular‐weight DNA isolation and long‐read sequencing on a Nanopore GridIONx5 sequencer of *Astrothelium subdiscretum* were done as described before for the lichen fungal culture of *Physcia stellaris* (Wilken et al., 2020).

### 2.3 Assembly, gene mining, and alignment

Raw Illumina short reads were checked with fastQC (https://www.bioinformatics.babraham.ac.uk/projects/fastqc/) before and after applying filtering with Trimmomatic (Bolger et al., 2014; LEADING:10 TRAILING:10 SLIDINGWINDOW: 4:15 MINLEN: 25). Filtered reads were assembled with SPAdes v3.14.0 (Bankevich et al., 2012; ‐k 21,33,55,77 ‐‐careful). Long reads of *A*. *subdiscretum* were assembled using a modified version of the “ont‐assemble‐polish” pipeline (https://github.com/nanoporetech/ont‐assembly‐polish). The pipeline used canu v1.8 (Koren et al., 2017) for the long‐read assembly with a genome size estimation of 39 megabases and racon v1.3.2 (Vaser et al., 2017) for scaffolding. Subsequently, the assembly was polished with the Illumina short reads of *A*. *subdiscretum* using Pilon v1.23 (Walker et al., 2014). All resulting assemblies were evaluated with QUAST v5.0.2 (Gurevich et al., 2013). The BUSCO v4.0.6 pipeline (Waterhouse et al., 2018) was then applied to the assemblies to retrieve single‐copy orthologous genes and benchmark the quality of the assemblies. Samples in dataset A were mined for orthologs using the Dothideomycetes BUSCO gene set, while samples in dataset B were mined using the Eurotiomycetes BUSCO gene set (orthologs used by BUSCO are from OrthoDB version 10; Kriventseva et al., 2019). A Python script (https://github.com/claudioametrano/BUSCO_2_alignments.py) was then used to build the alignment using only the orthologs present in each assembly of the dataset (A or B). MACSE v2 (Ranwez et al., 2018) was then applied to perform codon‐aware alignments. MACSE is integrated in a pipeline (https://github.com/ranwez/MACSE_V2_PIPELINES), which combines it with the segment‐filtering method HMMCleaner (Di Franco et al., 2019). The prefiltering and postfiltering methods of the MACSE pipeline were disabled (‐‐no_prefiltering; ‐‐no_postfiltering). The resulting gene alignments were then subjected to a second filtering step with the block‐filtering method using gBlocks v0.91 in codon version with relaxed parameters (Castresana, 2000; Talavera & Castresana, 2007). Randomly picked alignments (~10 each dataset) were manually inspected after each step.

In addition to protein‐coding genes retrieved by BUSCO, rDNA regions of each genome assembly were extracted. For each genome, the assemblies were aligned with blastn to the 18S, ITSRefSeq, and 28S fungal databases (BLAST v2.11.0+; https://www.ncbi.nlm.nih.gov). All identified scaffolds with rDNA regions were then aligned to sequences from the NCBI nucleotide database to delimit the specific rDNA region. For assemblies that only contained a partial rDNA region or without BLAST hits, rDNA regions were reconstructed using the raw reads with GRAbB (Brankovics et al., 2016). Incomplete rDNA sequences from the previous BLAST step or ribosomal markers of the same species from NCBI were used as bait to assemble complete rDNA regions. A further scaffolding step (when needed) and trimming of the poorly aligned positions were then carried out manually. GC content was calculated after the filtering steps both for protein‐coding and for rDNA alignments.

### 2.4 Molecular phylogeny

Nucleotide alignments of genes that were longer than 900 bp (300 codons), after filtering, were concatenated using FASconCAT‐G (Kück & Longo, 2014). The concatenated alignment was used to calculate a maximum‐likelihood tree with IQ‐TREE 2 (Minh et al., 2020) using the GTR+G substitution model. The fast‐bootstrapping option implemented in IQ‐TREE 2 was used to calculate 1000 bootstrap replicates. The phylogenetic relationship inferred (Figure S1) was used for the unrooted (pruned, in dataset B; Figure 1) constrained topology in subsequent rate analyses.

### 2.5 Rates of molecular evolution

Nucleotide substitution rates were measured in baseml or codeml (PAML v4.7e; Yang, 2007) using nucleotide and amino acid alignments longer than 900 bp/300 codons/300 amino acids. The topology was constrained on the base of the precalculated ML tree. In baseml, branch lengths were calculated for each nucleotide alignment separately with parameters: model = 7, Mgene = 0, clock = 0, fix_alpha = 0, Malpha = 0,ncatG = 10, and cleandata = 0. In order to partition the protein‐coding genes by codon position, the same alignments used in the previous analysis were converted to the phylip format to exploit the options G (multiple partitions in the alignment) and C (partition by codon position), together with Mgene = 1, which calculates a separate set of branch lengths for each partition (codon position). Ribosomal DNA markers (18S, ITSs with 5.8S and 28S) were analyzed by baseml using nucleotide substitution settings (see above) and partitioning the alignments by locus.

Amino acid alignments were used in codeml with: clock = 0, aaDist = 0, aaRatefile = BLOSUM62.dat, model = 2, and cleandata = 0. Codon model analyses were performed using the extension for codeml ete3 evol (ETE3; Huerta‐Cepas et al., 2016). ω is the ratio between non‐synonymous (d*N*) and synonymous substitution rates (d*S*). Nested branch models M0 (same *ω* for the entire tree), b_free (two *ω*), b_free (three *ω*), and fb (one *ω* each tree branch; free‐ratio model) (Yang & Nielsen, 2002) were run on each alignment to evaluate what model better fits our data and to estimate d*N*, d*S*, and *ω*. In the b_free (two *ω*) model, the lichenized clade and non‐lichenized clade have the same *ω*, but the outgroup has a different one. In the b_free (three *ω*) model, a different ω parameter was assigned to the outgroup and to the lichenized and non‐lichenized clade, respectively. Highly divergent sequences can easily lead to a saturation of synonymous substitution estimation; therefore, the data from the free‐ratio codon model were strictly filtered; only the genes with d*S* values lower or equal to three (d*S* ≤ 3) (Yang, 2014) were retained. In addition, all genes with *ω* > 10 were discarded (filtered dataset), since large *ω* values are very likely due to assembly or annotation errors that caused d*S* values to tend toward zero (Rubin & Moreau, 2016).

Branch‐site models bsA and bsA1 (null model) were applied (Zhang et al., 2005) using ete3 evol for codeml, to detect genes having codons under positive selection. These models were applied with a setting that defined the lichenized clade as foreground branches (the branches allowed to have a fraction of sites with *ω* > 0) and then repeated with a setting that defined the non‐lichenized clade as foreground branch. These settings allowed to identify genes that are assessed under positive selection exclusively for lichenized fungi. For genes having codons under positive selection in lichenized clades only, Gene Ontology (GO) terms were retrieved from OrthoDB (https://www.orthodb.org/).

From all PAML output files, long‐term evolutionary rates were calculated for each clade (lichenized, non‐lichenized) in the trees by averaging branch lengths from the tips to the common ancestor node in a tip‐to‐root fashion (Barraclough & Savolainen, 2001; Lanfear et al., 2010, 2013). This procedure was applied to both nucleotide (using codon or not) and amino acid rate estimations. This method of rate calculation was adopted to avoid the bias introduced when non‐independent samples are used in comparative analyses (Felsenstein, 1985). Since the sum of all branches (from the tips, i.e., the present species, to the common ancestor node) represents the same timespan for the considered clades, we did not calibrate the tree to obtain absolute substitution rates.

### 2.6 Statistical analysis

The distributions of gene rates and ω were compared in Prism 8.3.0 to assess global differences in genome evolutionary rates. Nonparametric test was selected after testing the normality of the distributions of gene rate with D'Agostino–Pearson and Shapiro–Wilk tests. Therefore, we applied the Wilcoxon matched‐pairs signed rank rest (nonparametric equivalent of the paired t test) to compare the distributions of averaged tip‐to‐root values of lichenized and non‐lichenized clades. The distributions of rates were paired by gene.

The four nested branch models and the two nested branch‐site models, used to test the presence of different selective pressure and positively selected genes, were compared in pairs by the likelihood‐ratio test (LRT) using a chi‐square distribution.

## 3 RESULTS

Genome assemblies were generated from Illumina short reads or Nanopore long reads for *Astrothelium subdiscretum* (Table 1, boldfaced). The total length of the assemblies was between 30 and 40 Mb and in line with the expected genome sizes for filamentous ascomycetes. Genome assembly statistics represented by the contig number, total assembly size, and the N50 value highlight good contiguity and a completeness of 89%–94% evaluated with BUSCO at the class level. The phylum‐level universal ortholog percentage was in the range of 94%–98% (Table 2). Using long‐read sequencing for *A*. *subdiscretum* resulted in a similar genome assembly as the genomes assembled from short reads. Particularly, the short read assembly of *P*. *massariospora* outperformed every other assembly (including the long‐read assembly of *A*. *subdiscretum*) and resulted in 41 contigs and an N50 value of 1,416,161 bp. Since most genomes assemblies were already in sufficient quality with only Illumina sequencing, we refrained from additional Nanopore sequencing for other fungal genomes than *A*. *subdiscretum*.

**TABLE 2:** Assembly statistics

Assembly name	Contigs No. (>1 kb)	Length Mb (>1 kb)	N50 (bp)	BUSCO % (Ascomycota)	BUSCO % (Dothideomycetes or Eurotiomycetes)
*Astrothelium macrocarpum*	611	36.4	200,777	C: 97.2 [S: 97.0, D: 0.2], F: 0.2, M: 2.6	C: 92.4 [S: 92.1, D: 0.3], F: 0.4, M: 7.2
*Astrothelium subdiscretum*	213	32.3	354,317	C: 97.7 [S: 97.1, D: 0.6], F: 0.2, M: 2.1	C: 94.1 [S: 93.4, D: 0.7], F: 0.4, M: 5.5
*Bathelium albidoporum*	977	32.7	88,197	C: 94.8 [S: 94.8, D: 0.0], F: 1.3, M: 3.9	C: 90.1 [S: 89.8, D: 0.3], F: 1.7, M: 8.2
*Pyrenula aspistea*	398	39.1	361,947	C: 96.4 [S: 96.2, D: 0.2], F: 0.3, M: 3.3	C: 91.8 [S: 91.6, D: 0.2], F: 0.6, M: 7.6
*Pyrenula massariospora*	41	37.9	1,416,161	C: 97.1 [S: 97.0, D: 0.1], F: 0.4, M: 2.5	C: 91.4 [S: 91.1, D: 0.3], F: 0.6, M: 8.0
*Trypethelium eluteriae*	1502	31.8	58,769	C: 93.7 [S: 93.6, D: 0.1], F: 2.6, M: 3.7	C: 89.3 [S: 89.0, D: 0.3], F: 2.4, M: 8.3

Note

Genome completeness is reported using the BUSCO output format (C: complete [S: single copy, D: duplicated], F: fragmented, M: missing). BUSCO benchmark uses 1706 genes for Ascomycota, and 3786 and 3546 genes for Dothideomycetes and Eurotiomycetes, respectively.

Gene models were extracted from these genome assemblies and filtered for the construction of two datasets. Dataset A was composed of 3786 orthologous genes commonly present in genomes from Dothideomycetes; 2569 of them were present in each of the 11 samples, and 1863 of them were included in the analyses being longer than 900 bp after the filtering steps. Dataset B was composed of 3546 orthologous genes commonly present in genomes from Eurotiomycetes; 2768 of them were present in each of the 9 samples, and 2085 of them were included in the analyses since they were longer than 900 bp after the filtering steps. Maximum‐likelihood phylogenies inferred from the concatenation of these genes provided the topology for the constrained trees; dataset A tree was used as it is, while dataset B tree was pruned to the tips actually used for each comparison (two lichenized fungi vs non‐lichenized fungi). Branch lengths of the ML tree inferred from the supermatrix were discarded (Figure 1) and recalculated for each marker. All nodes of the ML trees were fully supported (Figure S1), as expected for such a large supermatrix and a low number of tips.

The strict filtering was applied to exclude potentially saturated markers (d*S* ≤ 3, *ω* < 10) for the codon free‐ratio model. In dataset A, 193 genes were retained. Strict filtering retained in dataset B 156 genes of Pyrenulales and 242 genes of Verrucariales when compared to *Exophiala*–*Capronia* and 102 genes of Pyrenulales and 129 genes of Verrucariales when compared to *Knufia*–*Cladophialophora*.

**FIGURE 2:**
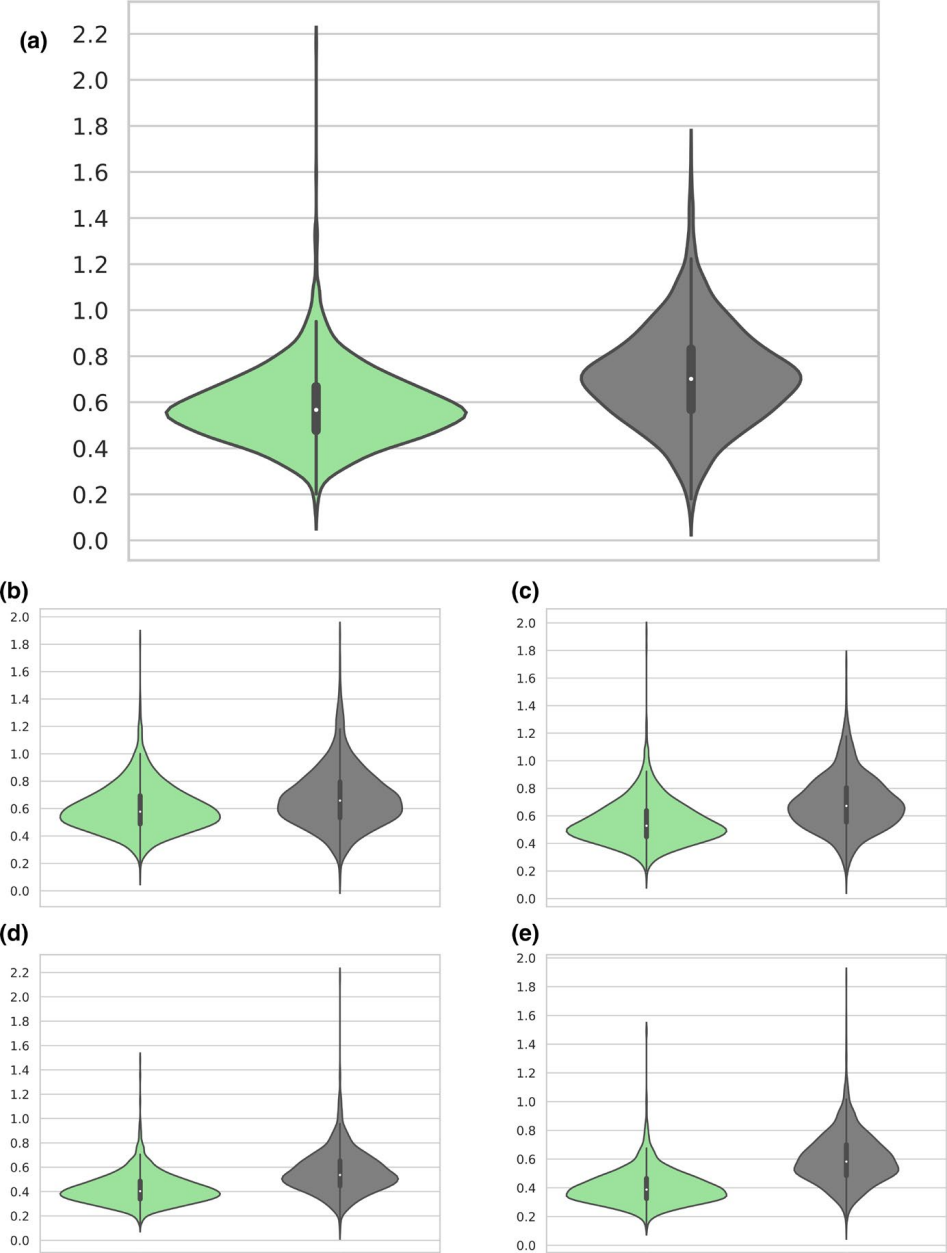
Tip‐to‐root nucleotide substitution rates (substitutions/site) distributions. Green violin plots represent lichenized samples, and gray violin plots represent non‐lichenized samples; median value is represented by the white dot, the black bar shows the interquartile range, black line shows lower/upper adjacent value, and violin shows the probability density of the distribution. (a) Dataset A (Dothideomycetes), (b) dataset B (Eurotiomycetes) Pyrenulales vs. *Exophiala sideris* and *Capronia epimyces*, (c) dataset B (Eurotiomycetes) Pyrenulales vs. *Knufia petricola* and *Cladophialophora psammophila*, (d) dataset B (Eurotiomycetes) Verrucariales vs. *E. sideris* and *C. epimyces*, and (e) dataset B (Eurotiomycetes) Verrucariales vs. *K. petricola* and *C. psammophila*

The lichenized clades consistently had lower substitution rates than the non‐lichenized clades when nucleotide, amino acid, or codon models were used on datasets A and B (Table 3). The median nucleotide substitution rates in both datasets A and B were significantly lower for lichens in every comparison performed (Wilcoxon's test, *p *< .0001; Table 3, Figure 2). The complete rate distributions in Figure 2 show higher density for lichenized clades at the median, as samples in these clades are more closely related than the samples in non‐lichenized clades; however, the range of the rate distributions is similar. While the majority of genes were slower evolving in the lichenized clades, 12.9%–26.3% of the analyzed genes showed a faster substitution rate (Table 3). In addition to the nucleotide substitution rates, we measured median values of the amino acid replacement rate, which were also significantly lower in the lichenized clades than in the non‐lichenized clades (Wilcoxon's test, *p *< .0001; Table 3, Figure S2). Furthermore, there were significantly slower substitution rates of the lichenized lineages in each codon position (Table 3), with *p *< .0001 for all the comparisons except one (Wilcoxon's test, *p *< .01). We also measured codon position rates in the strictly filtered dataset. When rate differences between lichen and non‐lichen genes occurred, the strict filtering of genes (about 5%–10% genes survived) determined a generalized decrease in the substitution values (of about 20%–50%), which was expected, as fast‐evolving genes (prone to saturation) were excluded. However, the filtering also determined a biased rate proportion between lichenized and non‐lichenized rates on each codon position (data not shown), but more apparent on third codon positions, which contributes the most to synonymous substitutions. Therefore, differences for third codon positions in filtered datasets were not always significant (Table 3). We also measured the nucleotide substitution rate of ribosomal markers (18S, ITSs with 5.8S and 28S), of which most were faster evolving in lichens contrary to our findings in most protein‐coding genes. Only the slower evolving 18S gene (0.019 subs/site) and ITS region (0.140 subs/site) rates in Verrucariales were slower when compared to the non‐lichenized clade rate (Table 3).

**TABLE 3:** Evolutionary rate median values and confidence interval, percentage of genes having higher substitution rate than the sister clade, and GC content

	Dataset A			Dataset B					
	Lichenized	Non‐lichenized	*p*	Lichenized Pyrenulales	Non‐lichenized (vs. Pyrenulales)	*p*	Lichenized Verrucariales	Non‐lichenized (vs. Verrucariales)	*p*
Nucleotide substitution rate	0.5669 (0.5596–0.5751)	0.7014 (0.6916–0.7111)	****	0.5779 (0.5724–0.5872) 0.5282 (0.5206–0.5347)	0.685 (0.6427–0.6674) 0.6722 (0.6634–0.6792)	**** ****	0.4035 (0.3981–0.4098) 0.3878 (0.3829–0.3932)	0.5365 (0.5295–0.5444) 0.5823 (0.5720–0.5918)	**** ****
Higher nucleotide substitution gene, %	26.3	73.7		18,9	81.1		12.9	87.1	
Amino acid replacement rate	0.2413 (0.2341–0.2478)	0.3037 (0.2970–0.3116)	****	0.2435 (0.2363–0.2503) 0.2380 (0.2306–0.2461)	0.2821 (0.2759–0.2887) 0.3199 (0.3141–0.3283)	**** ****	0.1865 (0.1825–0.1912) 0.1838 (0.1785–0.1885)	0.2594 (0.2532–0.2641) 0.3002 (0.2931–0.3068)	**** ****
*ω*	0.02531 (0.02383–0.02746)	0.007662 (0.007109–0.008197)	****	0.005273 (0.004507–0.006089) 0.008252 (0.006543–0.009881)	0.004909 (0.004452–0.005395) 0.004697 (0.004476–0.004972)	****	0.01332 (0.01222–0.01495) 0.01467 (0.01337–0.01619)	0.009718 (0.007707–0.01194) 0.005255 (0.005051–0.005488)	****
d*S*	4.528 (4.106–4.946)	26.66 (22.92–26.87)	****	25.72 (19.43–33.49) 13.46 (11.01–18.77)	39.42 (34.33–46.17) 35.99 (35.11–36.95)	**** ****	7.318 (6.528–8.218) 6.199 (5.651–6.680)	12.72 (10.28–16.42) 33.62 (32.74–34.46)	**** ****
d*N*	0.1307 (0.1272–0.1347)	0.1916 (0.1870–0.1953)	****	0.1319 (0.1285–0.1354) 0.1295 (0.1263–0.1325)	0.1542 (0.1512–0.1582) 0.1740 (0.1702–0.1789)	**** ****	0.1029 (0.1006–0.1060) 0.1016 (0.09900–0.1040)	0.1429 (0.1395–0.1459) 0.1644 (0.1617–0.1681)	**** ****
*ω* (filtered)	0.04457 (0.04006–0.05066)	0.05430 (0.05022–0.05785)	****	0.04722 (0.03830–0.05285) 0.03845 (0.02831–0.04923)	0.05009 (0.04536–0.05735) 0.04963 (0.04376–0.05519)	* ****	0.04157 (0.03618–0.04495) 0.03455 (0.02702–0.04089)	0.05620 (0.05140–0.06036) 0.04545 (0.03956–0.05285)	**** ****
d*S* (filtered)	1.588 (1.513–1.666)	2.097 (2.009–2.202)	****	1.872 (1.765–1.984) 1.752 (1.623–1.977)	1.987 (1.811–2.114) 1.862 (1.668–2.199)		1.706 (1.585–1.837) 1.730 (1.521–1.846)	1.894 (1.731–2.011) 1.912 (1.677–2.209)	**
d*N* (filtered)	0.07250 (0.06538–0.08528)	0.09965 (0.09448–0.1128)	****	0.08568 (0.06860–0.1025) 0.06568 (0.05500–0.08950)	0.0894 (0.07760–0.1055) 0.08435 (0.07515–0.09445)	**** ****	0.06675 (0.06090–0.07155) 0.05485 (0.04740–0.06200)	0.09705 (0.08535–0.1094) 0.08555 (0.07750–0.09145)	**** ****
1st codon position	0.2363 (0.2302–0.2421)	0.3177 (0.3100–0.3250)	****	0.2312 (0.2250–0.2370) 0.2278 (0.2209–0.2328)	0.2785 (0.2725–0.2847) 0.3282 (0.3210–0.3346)	**** ****	0.1811 (0.1760–0.1848) 0.1795 (0.1739–0.1843)	0.2514 (0.2448–0.2572) 0.3046 (0.2975–0.3109)	**** ****
2nd codon position	0.1387 (0.1348–0.1421)	0.1925 (0.1873–0.1971)	****	0.14 (0.1351 0.1442) 0.1368 (0.1335–0.1413)	0.1722 (0.1678–0.1764) 0.2006 (0.1956–0.2050)	**** ****	0.1055 (0.1030–0.1088) 0.1053 (0.1026–0.1086)	0.1546 (0.1499–0.1589) 0.1859 (0.1805–0.1897)	**** ****
3rd codon position	2.604 (2.539–2.705)	3.367 (3.256–3.458)	****	3.01 (2.872–3.133) 3.599 (3.390–3.796)	3.051 (2.945–3.178) 4.217 (3.997–4.455)	** ****	1.111 (1.089–1.133) 1.062 (1.043–1.078)	1.337 (1.305–1.363) 1.394 (1.372–1.426)	**** ****
1st codon position (filtered)	0.1408 (0.1277–0.1558)	0.1706 (0.1554–0.1923)	****	0.1482 (0.1231–0.1705) 0.1224 (0.1100–0.1465)	0.1822 (0.1489–0.1938) 0.1570 (0.1386–0.1763)	**** ****	0.1189 (0.1076–0.1317) 0.09866 (0.08937–0.1151)	0.1622 (0.1503–0.1821) 0.1530 (0.1402–0.1725)	**** ****
2nd codon position (filtered)	0.07875 (0.06659–0.08928)	0.1044 (0.09074–0.1131)	****	0.08712 (0.07676–0.1046) 0.06554 (0.05252–0.0788)	0.08955 (0.08089–0.1112) 0.09137 (0.07883–0.1050)	*** ****	0.06736 (0.06163–0.07682) 0.05830 (0.04873–0.07186)	0.1032 (0.09227–0.1169) 0.08925 (0.07874–0.1066)	**** ****
3rd codon position (filtered)	1.62 (1.436–1.854)	1.822 (1.665–2.208)	****	1.944 (1.644–2.240) 1.760 (1.562–2.094)	1.921 (1.641–2.206) 1.618 (1.312–2.107)	*	0.8769 (0.8299–0.9359) 0.05830 (0.04873–0.07186)	1.013 (0.9354–1.072) 0.08925 (0.07874–0.1066)	*** ****
Nucleotide substitution rate of rDNA 18S	0.081	0.032		0.056	0.028		0.019	0.025	
Nucleotide substitution rate of rDNA ITSs	0.68	0.27		0.22	0.17		0.14	0.17	
Nucleotide substitution rate of rDNA 28S	0.13	0.035		0.031	0.029		0.034	0.021	
GC content (%) of protein‐coding genes	51.6	55.2		51.3	52.8		51.2	52.8	
GC content (%) of rDNA	46.6	50.7		49	49.7		49.7	49.7	

Note

Median values and their 95% confidence interval in brackets. They are expressed in substitutions/site (nucleotide, amino acid, or codon). The filtered dataset only reports the value from the genes with d*S* < 3 and *ω* < 10. Where two values are reported (dataset B), two comparisons with different non‐lichenized samples were performed (see the Section 2). Asterisks highlight significant differences between lichenized and non‐lichenized clades by the Wilcoxon test (****p < .0001, ***p < .001, **p < .01, and *p < .05).

Branch codon models were tested pairwise by LRT (*p *< .01) (Table S1) to assess which model was able to fit best our data. The most parameter‐rich model fb (free‐ratio) was the one passing the LRT for the largest fraction of genes, when tested against the 2*ω* or M0 null models (M0‐fb and 2ω‐fb in Table S1). However, it provided a better fit only for a smaller fraction of genes when the null model already accounts for different ω parameters between lichenized and non‐lichenized lineages (3ω‐fb in Table S1). A comparison between the 2*ω* and 3*ω* models allowed to reject the null hypothesis for the majority of genes, except for the comparisons performed on dataset B (Pyrenulales) (2*ω*–3*ω* in Table S1). For these genes in dataset B, a simpler model using less *ω* parameters fitted better.

**FIGURE 3:**
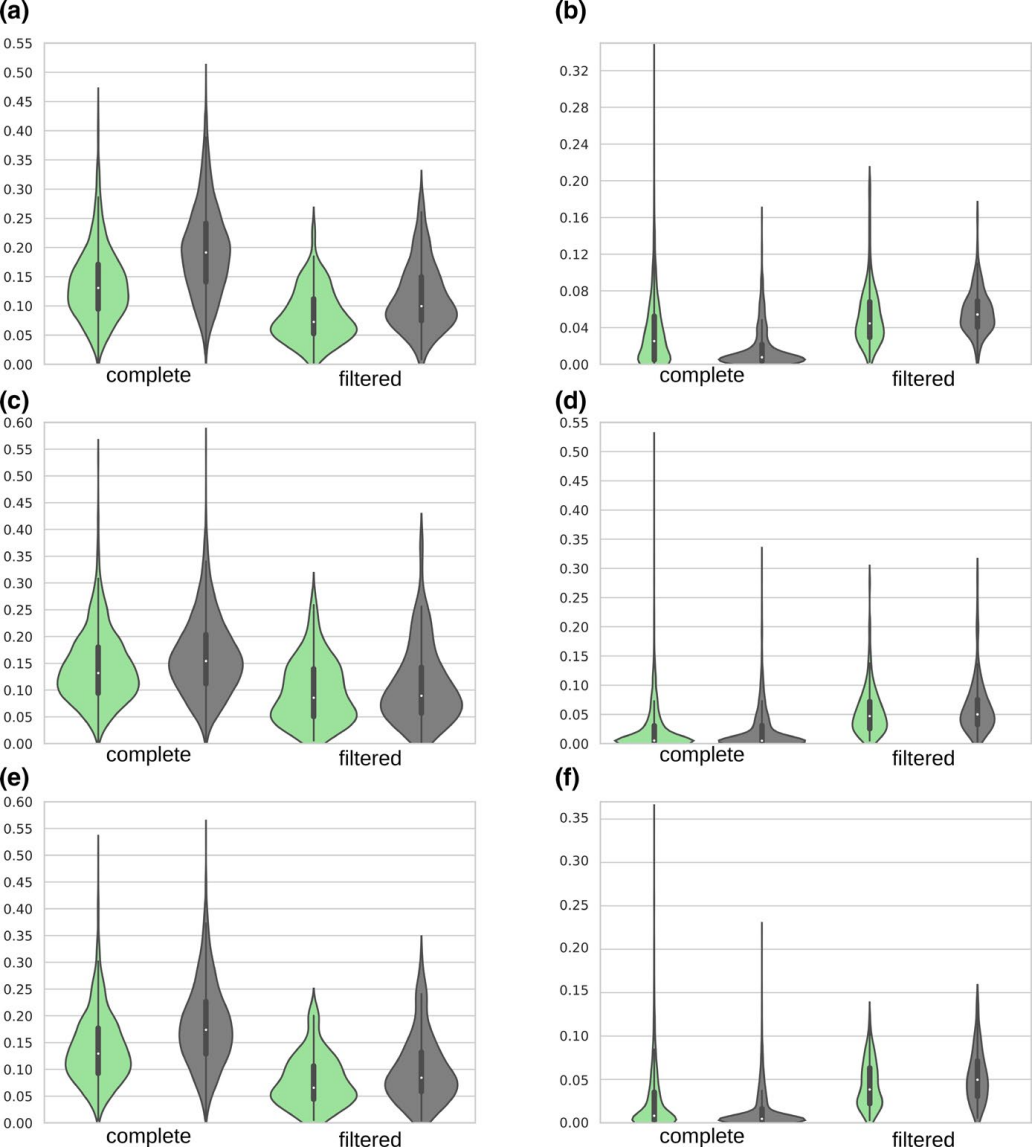
d*N* (a, c, e) (substitutions/site) and *ω* (b, d, f) distributions before (“complete”) and after (“filtered”) the strict filtering. Violin plots in a and b correspond to dataset A, c and d correspond to dataset B (Pyrenulales), and e and f correspond to dataset B (Verrucariales). Only one of the two comparisons performed for dataset B is reported (*K*. *petricola*, *C*. *Psammophila*), the *E. sideris*, and *C*. *epimyces* comparison is reported in Figure S3. Green plots represent lichenized lineages, and gray plots represent non‐lichenized lineages; median value is represented by the white dot, the black bar shows the interquartile range, black line shows lower/upper adjacent value, and violin shows the probability density of the distribution

Based on the results of the LRT, we chose the free‐ratio branch codon model to calculate d*S*, d*N*, and *ω* and to compare their distributions using the complete and strictly filtered datasets (Figure 3). d*S* and d*N* were significantly lower (Wilcoxon's test, *p* < .0001) in lichenized clades than in non‐lichenized clades (Table 3). In the strictly filtered datasets, the removal of most of faster evolving genes made the difference for synonymous substitutions not significant (Wilcoxon's test, *p* > .05) except for one of the comparisons in dataset B (*p *< .01). The removal of extreme d*S* values strongly influenced the estimation of *ω*, which is significantly higher for lichens in the complete datasets, and has instead lower median value when the strict filtering is applied (Table 3, Figure 3b,d,f). The filtering approach was applied to the free‐ratio codon model, as it is known as sensitive to substitution saturation on the third codon position (Yang, 2014); it was also used in nucleotide substitution model analysis, when dataset was partitioned by codon position.

Branch‐site codon models were then used to identify genes having sites under positive selection in lichenized and non‐lichenized clades. One gene of dataset B was inferred to be under positive selection when a lichenized clade was used as the foreground branch and when a non‐lichenized clade was used as the foreground branch. A higher number of genes were inferred to have sites under positive section when the lichenized clades were set as foreground. The LRT (*p *< .05) identified 16 genes in the lichenized clade having sites under positive selection and one for the non‐lichenized clade in dataset A. For dataset B, 12 or 20 genes were identified having sites under positive selection for Pyrenulales versus 7 or 6 for the non‐lichenized clades and 51 or 64 genes for Verrucariales versus 9 or 12 for the non‐lichenized clades. Often, the null model was rejected when a small fraction of the alignment sites was under neutral evolution (*ω* = 1), with *ω* never exceeding one at any site; in dataset A, this was the case for 11 out of 16 genes, and in dataset B, for 4 out of 12 and 11 out of 20 genes for Pyrenulales comparisons, and for 28 out of 51 and 34 out of 64 for Verrucariales. Moreover, among these genes with positively selected sites or with sites under neutral evolution, only a fraction (~20%) is consistently recovered when the background branch for the comparison was changed (dataset B). Among genes detected as positively selected, the most represented Gene Ontology (GO) molecular function terms were as follows: “transferase activity” (7 genes), “zinc ion binding” (5 genes), “ATP binding” (heat‐shock protein 70 family, 5 genes; protein kinase, 2 genes), “integral component of membrane” (5 genes), “transmembrane transport” (4 genes), and oxidoreductase activity (2 genes).

The GC content of protein‐coding genes and rDNA was lower in lichenized clades than in non‐lichenized clades (Table 3). Protein‐coding genes of non‐lichenized fungi had an average GC content of 54.03% compared with an average GC content of 51.34% of lichenized fungi. The rDNA region (18S, 28S, ITSs) of non‐lichenized fungi had an average GC content of 50.19% compared with an average GC content of 48.43% of lichenized fungi.

## 4 DISCUSSION

In this study, we detected overall slower evolutionary rates in a representative part of the protein‐coding genes from the genomes of two distantly related lineages of lichenized fungi, when compared to their non‐lichenized sister clades. Since these two lichenized clades evolved from independent lichenization events, these findings provide strong preliminary evidence of a convergence toward slower evolution, possibly under the influence of the symbiotic lifestyle and its ecological implications.

Fungi are well known for their ability to form diverse associations with photosynthetic organisms, to the point that a generalized, latent capacity of symbiosis between fungal and algal partners was verified for non‐symbiotic species (Hom & Murray, 2014). However, it is less clear how this peculiar lifestyle, once in place, can influence the evolution of fungi involved in mutualistic symbioses. Few studies directly investigated the possible consequences of a mutualistic lifestyle on evolutionary rates (Lutzoni & Pagel, 1997; Rubin & Moreau, 2016), highlighting relevant differences in substitution rates for mutualistic lineages. Only one of these investigations has been conducted to verify the possible connection between the switch to a lichenized lifestyle and an evolutionary rate change (Lutzoni & Pagel, 1997), although lichen symbiosis is a successful association, with almost 20% of currently known fungi adopting this lifestyle (Lumbsch & Rikkinen, 2017). Lutzoni and Pagel (1997) detected an increase in evolutionary rates in ribosomal markers for independent events of lichenization and concluded that lichenized fungi could have these elevated evolutionary rates due to higher UV exposure than in non‐lichenized relatives with subterraneous vegetative hyphae. We measured a lower GC content across protein‐coding genes and ribosomal regions in lichens, which may indicate a C‐to‐T mutation bias that could be caused by increased UV radiation (Ikehata & Ono, 2011) due to their exposed lifestyle. We also detected higher substitution rates in ribosomal regions, but as an exception to overall reduced rates in protein‐coding genes. This pattern of the ribosomal region not following the trend of the genome was also found for other organisms (Mitterboeck et al., 2016; Su & Hu, 2012), but it remains unclear why the ribosomal regions evolved differently than many protein‐coding genes (also when only considering the third codon position).

To determine whether our results based on protein‐coding genes can be classified under a broad definition of the Red King dynamic is not straightforward, given the theory is not completely settled on this topic, and also because our results on evolutionary rates do not contain any information about the relative benefits the bionts receive from being in a symbiosis (Bergstrom & Lachmann, 2003). In addition, Veller et al. (2017) identified several symbiosis classes in which slower evolution could be beneficial (i.e., the Red King). They assessed the impact of biological parameters such as generation time, mutation rate, selection strength, and population size in population models. The model indicated that mutation rate has a relevant role only for antagonistic symbiosis (Red Queen effect; i.e., faster evolution is more successful), but not for mutualist symbionts. However, it was also shown that depending if the mutualism has a small or large benefit for the bionts, evolutionary rate parameters such as longer generation time, lower selection strength, and smaller population size can have a short‐term and/or long‐term advantages for mutualistic symbioses. Some of these described evolutionary rate parameters leading to a Red King effect (i.e., slower evolution is more successful) may be also applicable to the discussion of the result we found for lichenized fungi.

The “universal” protein‐coding marker genes (BUSCO genes) used in this study are predictably under strict purifying selection (*ω* → 0) for both lichenized and non‐lichenized lineages. However, the complete dataset identified lichens as having a slightly less strict purifying selection (higher *ω*) acting on the genes we tested, which can be beneficial in some mutualistic symbiosis (Red Queen). The opposite trend was identified for the filtered dataset, which produced biased ω values, due to the drastically diminished sample size and the exclusion of extreme d*S* values (mostly present in non‐lichenized samples [data not shown]). However, completely different selection strengths (e.g., positive selection), acting on genomic regions other than the one studied, and involved in the establishment or functioning of the lichen symbiosis, cannot be excluded.

Our measurements are limited to two clades of lichenized fungi. In a general scenario of reduced evolutionary rates, these two clades of lichenized lineages had more genes with (few) sites subjected to positive selection, or neutrally evolving (*ω* = 1). However, the detection of such sites was consistent only for a small fraction of genes when lichenized clades were compared to a different sister clade. Therefore, the changed sites cannot be attributed with confidence to positive selection or neutral evolution acting on lichens. Moreover, these models are thought to lack detection power under synonymous substitutions saturation (Gharib & Robinson‐Rechavi, 2013), which was the case for the divergent sequences we used.

Lower evolutionary rates in lichenized fungi could be a consequence of the lichen biology and ecology. Lichens are thought to have long generation times, as indirectly confirmed by their generally low growth rates (Armstrong, 1983; Fortuna & Tretiach, 2018) and by direct estimations (Høistad & Gjerde, 2011). Lichen growth can be constrained by the carbon production of a relatively small population of algae (Scheidegger & Goward, 2002). But even in axenic cultures, where nutrient‐rich culture media are used, lichenized fungi often exhibit slow growth rates in comparison with many other filamentous ascomycetes with different lifestyles. Slow growth rates and longer generation times can provide a possible explanation for the lower substitution rates that we detected in lichenized clades and could have been contributed to the success and stability of the lichen symbiosis (Red King). Such an association between long generation time and slow evolutionary rate was also highlighted in other organisms (Welch et al., 2008) and in Ascomycota at a subphylum level (Shen et al., 2020). An attempt to assess a relationship between these two characteristics was made by Lanfear et al. (2013) who detected traits in plants that can influence their evolutionary rate. However, this study used measurements (e.g., plant height), which are unavailable to us for the lichens in this study. Although there are no such data for the species we used in this study, it is reasonable to think that most lichens have long generation times by low growth rates.

Another evolutionary rate parameter that can slow evolution and benefit the Red King effect is the size of a population. Population size can be estimated from genomic data. However, these population size estimations rely on multiple genomic samples belonging to the same species (or closely related species). The analyses are usually conducted on neutrally evolving sequences, which exclude coding regions (Gronau et al., 2011). Unfortunately, the number of samples included in this study did not allow reliable estimation of the population sizes. Moreover, the non‐lichenized groups available for comparisons were rich in lifestyles, such as pathogenic or parasitic lifestyles that could have a strong effect on evolutionary rates. In particular, pathogens are often subject to accelerated evolutionary rates as a result of the Red Queen dynamic (Papkou et al., 2019; Paterson et al., 2010). This limited our selection, and we had, for example, to exclude the recently described order Phaeomoniellales (Chen et al., 2015) from dataset B as it is mostly composed of phytopathogenic and endophytic species. An important aspect of studies on the Red King dynamic is the rate relationship between two bionts in the same symbiosis. For lichens, we currently lack information about the rates of the corresponding photosynthetic partners. The only experimental data about relative rates in lichens were provided by Zoller and Lutzoni (2003) who verified higher rDNA substitution rates in mycobiont *Omphalina*, a basidiolichen, when compared to its photobiont *Coccomyxa*. Since we focused on the genomic evolution of lichenized fungi in this study, we only sequenced mycobiont cultures. Future studies should include the photobionts to allow an investigation of relative rates in the lichen symbiosis.

Despite some limitations, our analyses provided the first evidence of slower evolutionary rates of lichen mycobiont genomes. This shift in evolutionary rates was often hypothesized for lichens, but never tested. Given the limited sampling this study allowed, further research involving other lichenized lineages, and other symbiotic systems (e.g., mycorrhizae) will be necessary to generalize this possible convergence toward slower evolution. This empirical evidence provides nevertheless important initial insights on how biotic cooperative interactions can shape the evolution of symbiotic organisms.

## CONFLICT OF INTEREST

The authors have no conflict of interest to declare.

## AUTHOR CONTRIBUTIONS

**Claudio G. Ametrano:** Data curation (equal); Investigation (equal); Methodology (equal); Writing – original draft (equal); Writing – review & editing (equal). **H. Thorsten Lumbsch:** Conceptualization (equal); Supervision (lead); Writing – review & editing (equal). **Isabel Di Stefano:** Investigation (equal). **Ek Sangvichien:** Investigation (equal). **Lucia Muggia:** Resources (equal); Writing – review & editing (equal). **Felix Grewe:** Conceptualization (lead); Investigation (equal); Writing – original draft (equal); Writing – review & editing (lead).

## Supporting information

Supplementary Material

## Data Availability

The data underlying this article have been deposited at DDBJ/ENA/GenBank under the accessions: JAGFMW000000000, JAGFMI000000000, JAGFMJ000000000, JAGFMK000000000, JAGFML000000000, and JAGFVP000000000. Assembly accessions are reported in Table 1. Multiple sequence alignments are available at https://doi.org/10.5281/zenodo.4609320. Lichen‐forming fungus cultures are available at the TISTR Culture Collection (Bangkok MIRCEN) with the TISTR Numbers: *Astrothelium macrocarpum* NSR6, *Astrothelium subdiscretum* UBN165, *Bathelium albidoporum* NSR34, *Trypethelium eluteriae* NAN5, *Pyrenula aspistea* KRB14, and *Pyrenula massariospora* TSL107.
