# Supplementary material for: Should we hail the Red King? Evolutionary consequences of a mutualistic lifestyle in genomes of lichenized ascomycetes
Source: Ecol Evol. 2022 Jan 11;12(1):e8471. doi: 10.1002/ece3.8471 (PMC8809443; doi:10.1002/ece3.8471)
Supplement: Supplementary file 1 — Supplementary Material [file ECE3-12-e8471-s001.docx]

**Supplemental Information for:**

**Should we hail the Red King? Evolutionary consequences of a mutualistic lifestyle in genomes of lichenized ascomycetes**

Claudio G. Ametrano, H. Thorsten Lumbsch, Isabel Di Stefano, Ek Sangvichien, Lucia Muggia, Felix Grewe

**Table of Contents:**

| **Figure S1** | Page 2 |
| --- | --- |
| **Figure S2** | Page 3 |
| **Figure S3** | Page 4 |
| **Table S1** | Page 5 |


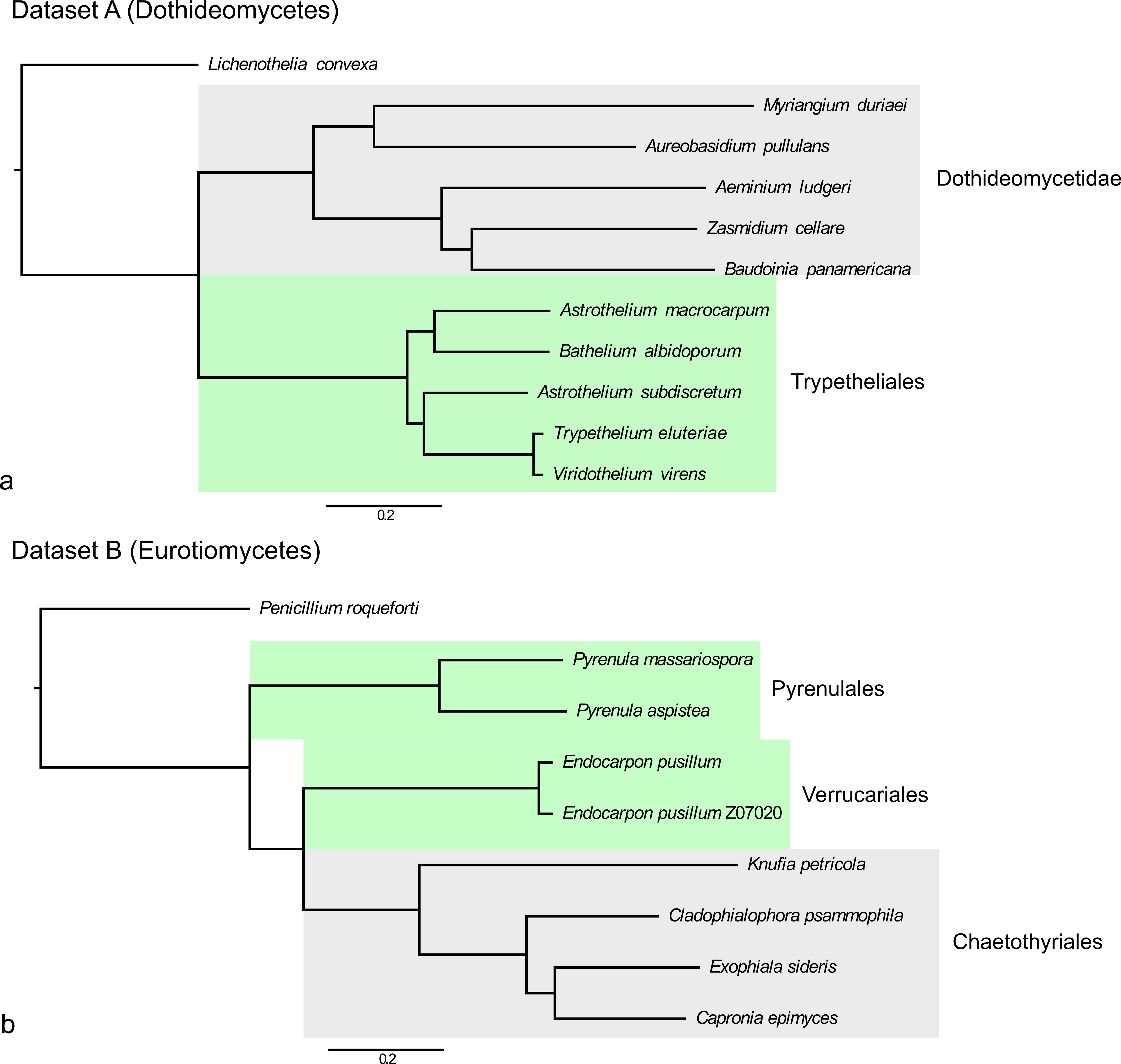


**Fig S1. Phylogenetic relationships of the samples** in Dataset A (**a**) and B (**b**). Green and gray boxes highlight lichenized and non-lichenized clades, respectively. Ultra-fast bootstrap shows full support for the entire tree.


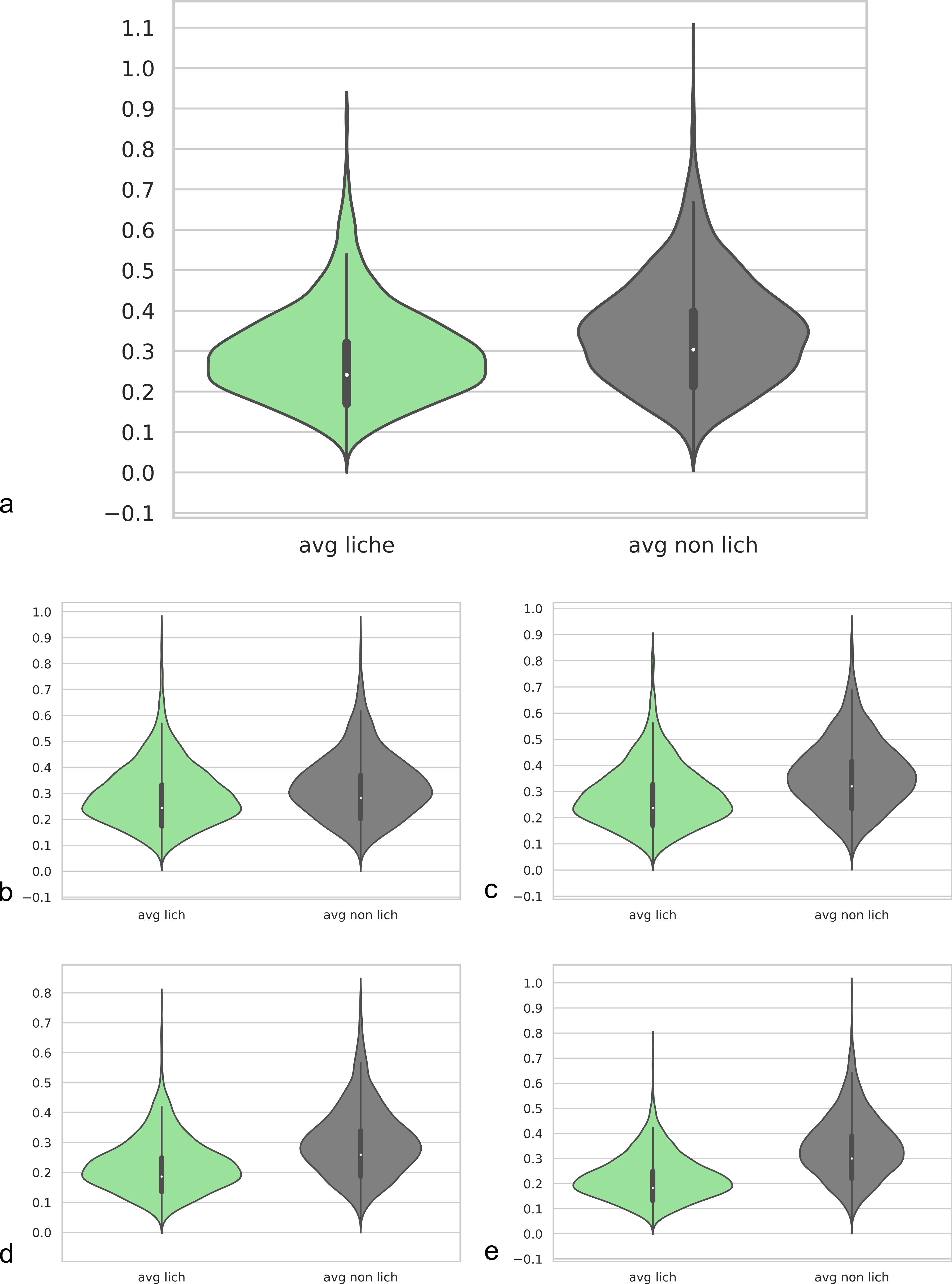


**Figure S2. Root-to-tips amino acid replacement rates** **distributions** (substitutions/site). Median value is represented by the black line, the box shows the interquartile range, wiskers at 10^th^ and 90^th^ percentile. (**a**) Dataset A (Dothideomycetes) (**b, c, d, e**) Dataset B (Eurotiomycetes).


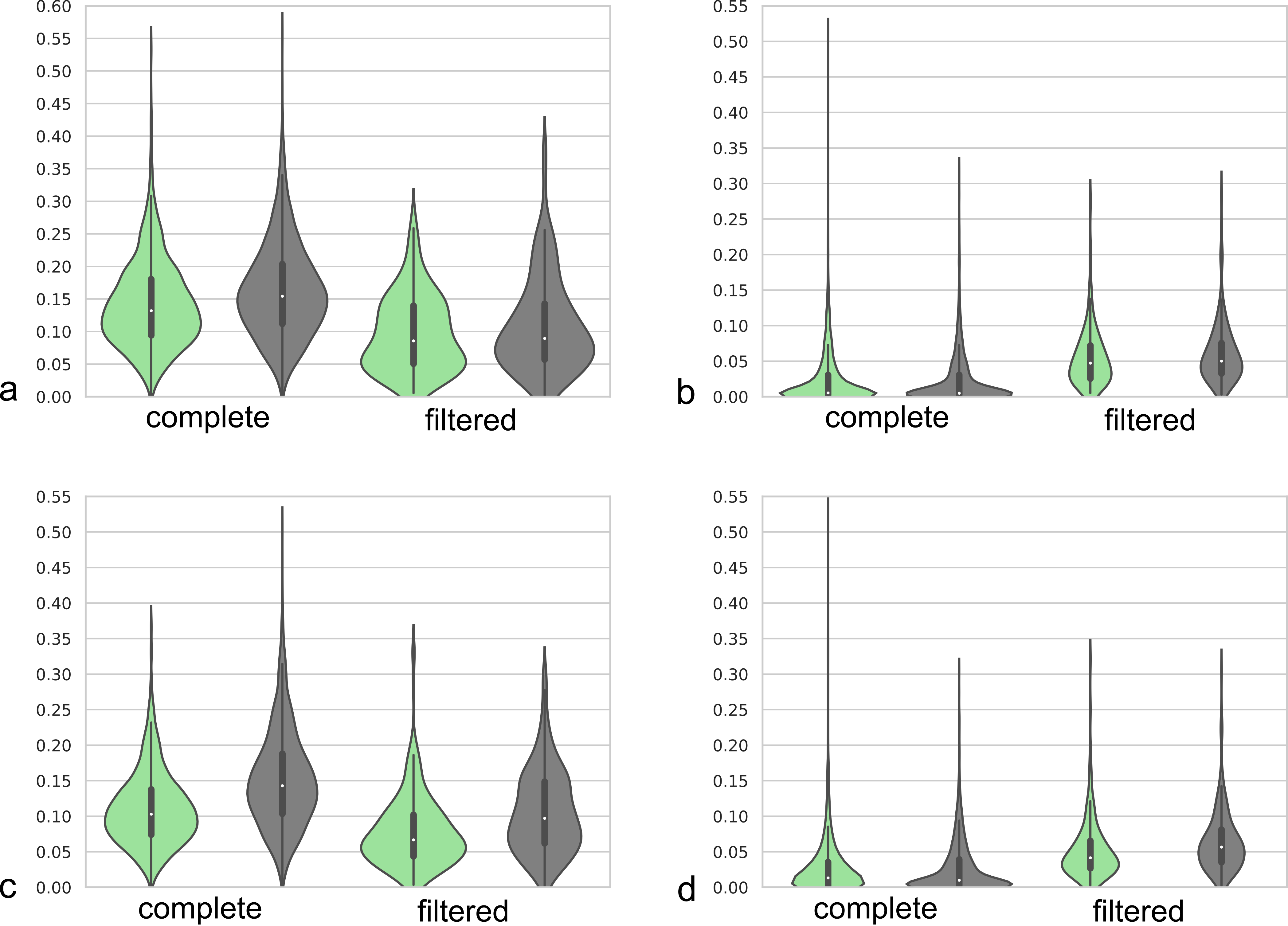
**Figure S3. Non-synonymous substitutions and omega.** dN (a, c) (substitutions/site) and ω (b, d) distributions before (“complete”) and after (“filtered”) the strict filtering. Violin plots in a and b correspond to dataset B (Pyrenulales), c and d correspond to dataset B (Verrucariales). Non-lichenized samples used here are *C*. *epimyces and E*. *sideris.*

**Table S1.** **Fraction of the genes that passed likelihood ratio test (LTR).**

| Dataset | Compared clades (with topology) | M0-2ω | M0-3ω | 2ω-3ω | M0-fb | 2ω-fb | 3ω-fb |
| --- | --- | --- | --- | --- | --- | --- | --- |
| A | (Trypetheliales, (Myriangiales, (Dothideales, Capnodiales))) | 0.23 | 0.77 | 0.74 | 0.84 | 0.82 | 0.42 |
| B | (Pyrenulales, (*Capronia*, *Exophiala*) | 0.35 | 0.44 | 0.20 | 0.48 | 0.31 | 0.21 |
|  | (Pyrenulales, (*Knufia*, *Cladophialophora*)) | 0.27 | 0.53 | 0.44 | 0.46 | 0.34 | 0.06 |
| B | (Verrucariales, (*Capronia*, *Exophiala*)) | 0.47 | 0.67 | 0.49 | 0.75 | 0.63 | 0.40 |
|  | (Verrucariales, (*Knufia*, *Cladophialophora*)) | 0.40 | 0.78 | 0.72 | 0.75 | 0.67 | 0.18 |

M0-2ω, M0-3ω, 2ω-3ω, M0-fb, 2ω-fb and 3ω-fb show which branch models were tested pairwise. Compared clades column shows the tree topology used for comparisons (without the outgroup).
